# Neuroprotective Effects of Sesamin and Sesamolin on Gerbil Brain in Cerebral Ischemia

**Published:** 2006-09

**Authors:** Fu-Chou Cheng, Tzyy-Rong Jinn, Rolis C. W. Hou, Jason T. C. Tzen

**Affiliations:** 1 *Stem Cell Medical Research Center, Department of Medical Research, Taichung Veterans General Hospital, Taichung, Taiwan (ROC);*; 2 *Graduate Institute of Biotechnology, National Chung-Hsing University, Taichung, Taiwan (ROC)*

**Keywords:** cerebral ischemia, neuroprotection, nitric oxide, sesamin, sesamolin

## Abstract

Sesamin and sesamolin, abundant lignans found in sesame oil, have been demonstrated to possess several bioactivities beneficial for human health. Excess generation of nitric oxide in lipopolysaccharide-stimulated rat primary microglia cells was significantly attenuated when they were pretreated with sesamin or sesamolin. The neuroprotective effect of sesamin and sesamolin was also observed *in vivo* using gerbils subjected to a focal cerebral ischemia induced by occlusion of the right common carotid artery and the right middle cerebral artery. Repeated treatment of sesamin or a crude sesame oil extract containing both sesamin and sesamolin significantly reduced the infarct size, visualized via 2,3,5-triphenyltetrazolium chloride staining, by approximately 50% when compared with the control group. These results suggest that sesamin and sesamolin exert effective neuroprotection against cerbral ischemia.

## INTRODUCTION

Cerebral ischemia resulted from low oxygen and glucose supply evidently decreases the formation of ATP (1-3). Various ATP-driven membrane-bound pumps or reuptake processes that usually work to maintain the homeostasis of important metabolites or ions become retarded, and moderate to severe neuronal damage may occur following ischemic events. Several models of focal ischemia have been developed to study the pathology and treatment of ischemic stroke in different animal species. In one widely used technique, cauterization or clipping of the middle cerebral artery in mice or gerbils through a subtemporal craniectomy is generally accepted as a valid experimental model of cerebral ischemia (4-6).

Seasame seeds and sesame oil have long been used as health foods in Asia to increase energy and to prevent aging. The nonfat portion (1-2%) of this oil contains sesamin, sesamol, sesamolin, sesaminol, and episesamin (7). Sesamin, the major lignan found in sesame oil has been demonstrated to enhance hepatic detoxification, to protect against oxidative stress, and to prevent the development of hypertension (8). Chronic ingestion of sesamin and vitamin E attenuates the elevation of blood pressure, oxidative stress and thrombotic tendency, suggesting that these treatments are presumably beneficial for the prevention of hypertension and stroke (9). Dietary sesame lignans reduce arachidonic acid content and the n-6/n-3 ratio in the liver, and promote ketogenesis in company with the reduction of polyunsaturated fatty acid esterification into triglycerides (10). These findings strongly suggest that sesame lignans are useful as a prophylactic treatment in the development of hypertension and cardiovascular hypertrophy.

In a previous study, sesamin and sesamolin were found to significantly quench the excess generation of nitric oxide (NO) induced by lipopolysaccharide (LPS) in the murine microglial cell line BV-2 and rat primary microglia cells (11). In this study, we firstly examined if the same neuroprotective effect could be observed when rat primary microglia cells were pretreated with sesamin or sesamolin prior to the stimulation of LPS. Furthermore, we tested if neuroprotection of gerbil brain cells in cerebral ischemia could be achieved by oral administration of the sesame lignans.

## MATERIALS AND METHODS

### Preparation of sesamin and sesamolin

Sesame oil of 200 mL was dissolved in 1500 mL acetone and stored at -70°C for 24 h. The solidified triacylglycerol was discarded and the acetone extract was collected after filtration. After acetone evaporation, the oil was saponified with 25 mL ethanol containing 5% potassium hydroxide for 1 h. The unsaponifiables were added with 100 mL distilled water and extracted three times with 60 mL diethyl ether. After ether evaporation, the unsaponifiables were added with 10 mL diethyl ether and air-dried at room temperature overnight. Crude extract of approximately 2 g was obtained as white crystalline powder containing 90% sesamin and 10% sesamolin. The crude extract was dissolved in 2 mL of chloroform and developed in a preparative thin-layer chromatography with a solvent system containing chloroform:ethanol (9:1) to separate sesamin and sesamolin (R_f_ 0.58 and 0.69, respectively). Purity of sesamin and sesamolin was analyzed by HPLC according to the method described by Kamal-Eldin *et al*. (12).

### Isolation of rat primary microglia cells

Rat primary microglia cells were isolated from glial cultures prepared as described by Hou *et al*. (11). Briefly, glial cells were cultured in 75 mm^2^ flasks for 10-14 days in Dulbecco’s modified Eagle’s medium (DMEM) supplemented with 10% fetal culf’s serum (Hyclone, Logan, UT, USA) containing 100 U/mL penicillin and 100 g/mL streptomycin. To separate the microglia, the flasks were shaken for 1 h at 240 rpm in a rotary shaker at 37°C. Detached cells were plated into 24 well plates at a density of 2 × 10^5^ cells per well. After 2 h of incubation at 37°C, nonadherent cells were removed and fresh medium was added. The purity of microglial cultures was assessed by using OX-42 antibody (PharMingen, San Diego, CA, USA), and more than 95% of cells were stained positively. Cells were washed twice with warm DMEM (without phenol red) and then cultured in the serum-free medium.

### LPS stimulation of microglia cells pretreated with sesamin or sesamolin

To evaluate if pretreatment of sesamin and sesamolin possessed potential neuroprotective activity, a preliminary test was performed to detect their inhibitory capabilities on NO level of rat microglia cells stimulated by LPS (*Escherichia coli* serotype 0111:B4 from Sigma, St. Louis, MO, USA). In this test, cells were incubated with sesamin or sesamolin, dissolved in dimethyl sulfoxide (DMSO) with the final concentration of this solvent added to the cell cultures never exceeding 0.1%, for 1 h prior to the supplement of LPS. All the cell culture ingredients were purchased from Life Technologies (Grand Island, NY, USA).

### Detection of NO production

NO production was assayed by measuring the level of nitrite, the stable NO metabolite, in the conditioned medium (11). Briefly, 100 L of culture supernatant was reacted with an equal volume of Griess reagent (1 part of 0.1% naphthylethylenediamine and 1 part of 1% sulfanilamide in 5% H_3_PO_4_) in 96-well tissue culture plates for 10 min at room temperature in the dark. The absorbance at 540 nm was recorded by a microplate reader (spectra MAX 340, Molecular Devices, Sunnyvale, CA, USA).

### Cerebral ischemia of gerbils

Eighteen male gerbils (60-85 g) were randomly divided into three groups fed by regular meal supplemented (op., 20 mg/kg/day), respectively, with saline (control), purified sesamin, and the crude sesame oil extract containing 90% sesamin and 10% sesamolin. After feeding for 4 days, each gerbil was anesthetized with chlorohydrate (400 mg/kg) intraperitoneally and its body temperature was maintained at 37°C with a heating pad (CMA/150). A midline neck incision was made and the right carotid artery was exposed and separated from the vago-sympathetic trunk. The right carotid artery was loosely encircled with a 4-0 suture for later occlusion. The gerbil’s head was placed in a stereotaxic frame (David Kopf, CA, USA) with the nose bar positioned 4.0 mm below the horizontal line. Following a midline incision, the skull was partially removed to expose the right middle cerebral artery. The middle cerebral artery was loosely encircled with an 8-0 suture for later occlusion. A focal cerebral ischemia was induced by occlusion of the right common carotid artery (CCA) and the right middle cerebral artery (MCA) for 60 min, followed by reperfusion for 3 h. MBF 3D, a laser probe (0.8 mm in diameter) of a Laser Doppler Blood Flow monitor (Moor Instruments, Axminster, England) was positioned onto the cortex with its tip close to the middle cerebral artery. Cerebral blood flow dropped to less than 5% of basal after the occlusion of the MCA. Cerebral blood flow reached its minimal level within 5 min after the start of the occlusion and was confirmed to remain at this level throughout the monitoring period to ensure the validity of the stroke model.

### Visualization of infarct size in gerbil brain

Approximately 24 h after cerebral ischemia, each gerbil was anesthetized and perfused transcardially with 2% isotonic heparinized saline and 2,3,5-triphenyltetrazolium chloride (TTC). The brain was then removed and sliced into five 2-mm-thick coronal sections for TTC staining as described by Bederson *et al*. (13). The brain slices were placed in 10% buffered formalin in the dark and then refrigerated until photographed. Infarct size was quantified by weighing the traced normal and infracted areas. All TTC data were analyzed by ANOVA with Student t tests, and *P*<0.05 was considered to be statistically significant.

## RESULTS

### Attenuation of NO production by sesamin and sesamolin in LPS-stimulated microglia cells

**Figure 1:**
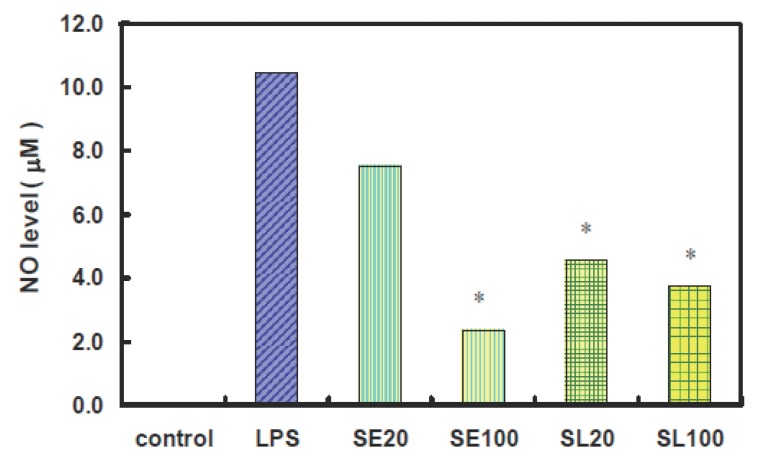
Attenuation of NO Production in LPS-Stimulated Microglia Cells by Sesamin and Sesamolin. Rat primary microglia cells were incubated with 20 or 100 μM of sesamin (SE) or sesamolin (SL) for 1 h, and then challenged with 1000 ng/mL LPS. Statistical significance (*P*<0.05) is indicated by a star.

Microglia cells have been demonstrated to be the major source of inflammatory factors that mediated LPS-induced neurotoxicity in neuron-glia cultures (14). Therefore, rat primary microglia cells pretreated with sesamin or sesamolin were subjected to LPS treatment, and the induced NO release was detected to evaluate whether sesamin and sesamolin could confer neuroprotective effects. The results show that excess generation of NO in the LPS-stimulated microglia cells was inhibited by pretreatment of sesamin or sesamolin in a dose-dependent manner (Fig. 1). For a significant attenuation of NO production in LPS-stimulated microglia cells, sesamolin seemed to be more potent than sesamin.

### Neuroprotective effects of sesamin and sesamolin on gerbils in cerebral ischemia

**Figure 2:**
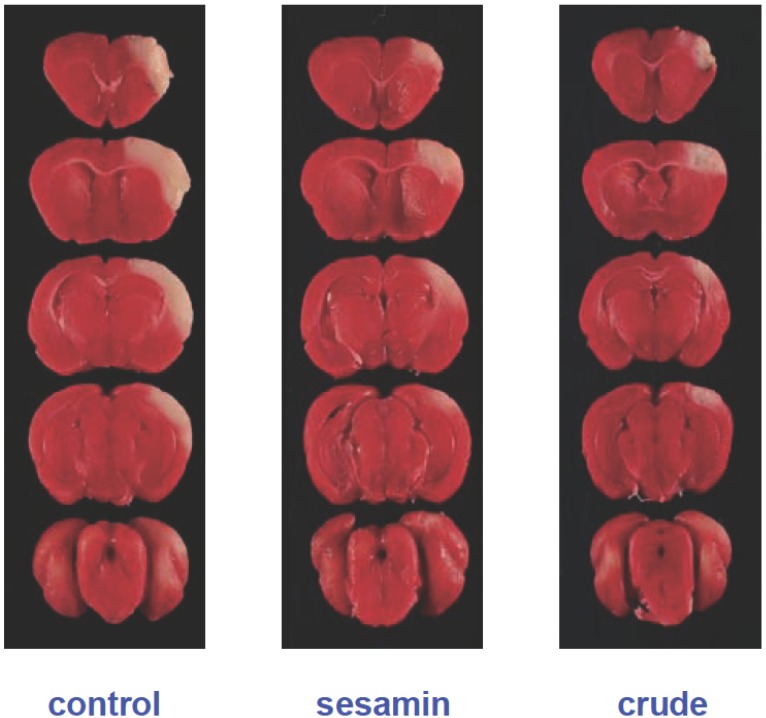
TTC Staining of Infarct Sizes of Gerbil Brains Caused by Cerebral Ischemia. Infarct sizes caused by cerebral ischemia were visualized by TTC staining in brains of gerbils pretreated with purified sesamin or the crude sesame oil extract containing both sesamin and sesamolin (20 mg/kg/day). Alive cells were stained in red while the damaged cells were unstained and visualized as white areas.

**Figure 3:**
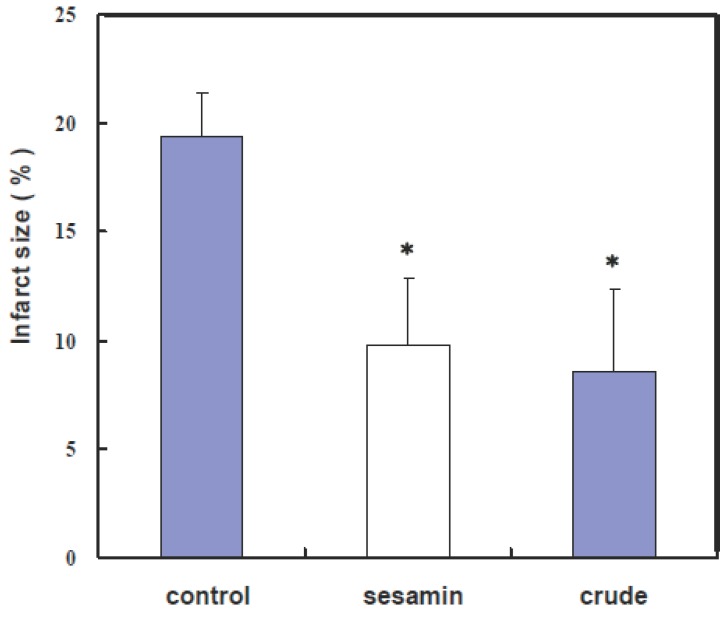
Neuroprotective Effect of Sesamin and the Crude Sesame Oil Extract on Infarct Sizes of Gerbil Brains in Cerebral Ischemia. Infarct sizes of gerbil brains shown in Figure 2 were quantified as described in Materials and Methods. Data are expressed as mean ± SEM. Statistical significance (*P*<0.05) is indicated by a star.

The observation of the neuroprotective effect of sesamin and sesamolin in the LPS-stimulated microglia cells provoked an inquiry if these sesame lignans could act as anti-ischemic agents *in vivo*. To examine this possibility, gerbils were repeatedly administered with sesamin or a crude sesame oil extract containing both sesamin and sesamolin prior to a focal cerebral ischemia. All the treated animals were found to have infarction in the cortex and caudate-putamen. Mean total infarct sizes, visualized by TTC staining (Fig. 2), in the three groups (control, purified sesamin, and crude sesame oil extract) were 91.9 mm^3^ (19.4 ± 2.0%), 46.5 mm^3^ (9.8 ± 3.1%), and 40.2 mm^3^ (8.5 ± 3.9%), respectively (Fig. 3). These results indicate that pretreatment with sesamin or the crude sesame oil extract for four days significantly reduced infarct sizes of gerbil brains in cerebral ischemia by 56% and 49%, respectively (*p*<0.05).

## DISCUSSIONS

In general, the occlusion of MCA and CCA does not always result in a predictable infarction volume. Collateral pathways in different species may redistribute blood flow sufficiently to prevent infarction in particular regions. In rats, cats and dogs, a simultaneous occlusion of a unilateral MCA and CCA is known to result in ischemic infarcts of variable size and degree. In rats, there is a need to occlude both the right and left CCA and one of MCA to produce predictable infarctions. However, the gerbil brain lacks the connection between the carotid and vertebrobasilar circulation resulting in an incomplete circle of Willis. Therefore, the occlusion of unilateral CCA and MCA in gerbils was proposed in the present study. Results of this study have demonstrated that a consistent infarct volume (19.4 ± 2.0%) could be achieved with severe infarction in both the cortex and caudate-putamen 24 h after the occlusion of unilateral CCA and MCA in gerbils. Our results were less severe but consistent with previous studies that utilized MCA occlusion or endovascular sutures (4-6). The experimental models of cerebral ischemia developed by Baskaya *et al*. (4), Yamamoto *et al*. (5), and Yoshimine *et al*. (6) were successful because of an incomplete anastomosis in gerbils. Advantages of the present model include simple craniectomy procedures, reproducible regional cerebral blood flow and consistent infarct volume.

Repeated pretreatment with sesamin or the crude sesame oil extract containing both sesamin and sesamolin for four days significantly reduced infarct sizes by approximately 50% in the present study. Although the mechanism of neuroprotection is not understood, the combined results of *in vitro* and *in vivo* experiments suggest that a reduction of neuronal injury may be related to an inhibition of the induced NO release when the brain produces free radicals during cerebral ishemia at a rate sufficient to overcome endogenous antioxidant defenses. It has also been proposed that sesamin and sesamolin may act as free radical-scavengers and inhibitors of lipid peroxidation (8). The neuroprotective efficacy of sesamin and sesamolin may also reflect their ability to scavenge free radicals over-produced during cerebral ischmia.

In conclusion, oral administration of sesamin or the crude sesame oil extract was neuroprotective in terms of reducing ischemic damage in experimental MCA+CCA occluded gerbils. The present results provide experimental data to sustain the potency of sesamin and sesamolin for neuroprotection against hypoxia or brain damage. Further investigation of optimal dosages of sesamin, sesamolin and the crude sesame oil extract are warranted in the future. Moreover, it is noteworthy that the crude sesame oil extract and the pure form of sesamin were equally effective in providing neuroprotection in this study. The pure form of sesamin requires many steps in its purification that leads to a relatively low recovery yield. Since the crude sesame oil extract demonstrates the same neuroprotective effect as the purified sesamin, this crude sesame oil extract seems to possess market potential as a supplement in health food products.
